# Environmental barriers to sociality in an obligate eusocial sweat bee

**DOI:** 10.1007/s00040-018-0642-7

**Published:** 2018-07-04

**Authors:** P. J. Davison, J. Field

**Affiliations:** 1 School of Life Sciences, University of Sussex, John Maynard Smith Building, Brighton, BN1 9QG UK; 2 Centre for Ecology and Conservation, University of Exeter, Penryn Campus, Cornwall, TR10 9EZ UK

**Keywords:** Sweat bee, *Lasioglossum*, Field transplant, Eusocial

## Abstract

Understanding the ecological and environmental contexts in which eusociality can evolve is fundamental to elucidating its evolutionary origins. A sufficiently long active season is postulated to have been a key factor facilitating the transition to eusociality. Many primitively eusocial species exhibit an annual life cycle, which is thought to preclude the expression of eusociality where the active season is too short to produce successive worker and reproductive broods. However, few studies have attempted to test this idea experimentally. We investigated environmental constraints on the expression of eusociality in the obligate primitively eusocial sweat bee *Lasioglossum malachurum*, by transplanting nest foundresses from the south to the far north of the United Kingdom, far beyond the natural range of *L. malachurum*. We show that transplanted bees can exhibit eusociality, but that the short length of the season and harsher environmental conditions could preclude its successful expression. In one year, when foundresses were transplanted only after provisioning first brood (B1) offspring, workers emerged in the north and provisioned a second brood (B2) of reproductives. In another year, when foundresses were transplanted prior to B1 being provisioned, they were just as likely to initiate nesting and provisioned just as many B1 cells as foundresses in the south. However, the life cycle was delayed by approximately 7 weeks and nests suffered 100% B1 mortality. Our results suggest that short season length together with poor weather conditions represent an environmental barrier to the evolution and expression of eusociality in sweat bees.

**Electronic supplementary material:**

The online version of this article (10.1007/s00040-018-0642-7) contains supplementary material, which is available to authorized users.

## Introduction

Determining how extrinsic environmental factors can affect the formation and persistence of social groups is critical to understanding the origin of complex social behaviours such as eusociality (Korb and Heinze 2008). The environment is thought to influence the geographic distribution and expression of social behaviour across a variety of vertebrate and arthropod taxa (Jetz and Rubenstein 2011; Purcell 2011; Faulkes and Bennett 2013; Sheehan et al. 2015, but see for example Gonzalez et al. 2013). Harsher or more variable environments might favour social behaviour because independent nest founding is risky, or because the presence of multiple individuals can act as a buffer against challenging or unpredictable conditions (Jetz and Rubenstein 2011; Hoiss et al. 2012; Kocher et al. 2014; Sheehan et al. 2015).

Eusociality is characterised by cooperative brood care and a reproductive division of labour (Wilson 1971), and considerable attention has been given to elucidating its evolutionary origins (see Bourke 2011). A perennial life cycle, nest thermoregulation, large colony size and food storage are derived characteristics of advanced eusocial insects such as ants and honeybees thought to favour eusociality in harsh environments (Kaspari and Vargo 1995; Hoiss et al. 2012; Wcislo and Fewell 2017). However, primitively eusocial bees and wasps, which lack morphological castes, and their solitary ancestors, typically complete an annual life cycle, form small groups and exhibit only limited nest thermoregulatory control (Cowan 1991; Reeve 1991; Potts and Wilmer 1997; Michener 2007). Thus, for primitively eusocial groups, eusociality may provide less buffering against environmental unpredictability. For example, workers can increase the chances of successfully rearing brood (e.g. Brand and Chapuisat 2014) but are of little value in years when conditions are so poor that offspring production is precluded altogether (Packer et al. 1989). Indeed, an annual colony cycle suggests both that the active season must be sufficiently long to sequentially produce workers and reproductives (Kocher et al. 2014), and that eusociality is inherently risky if any reproductive payoff is delayed until the end of the season (Fu et al. 2015). Consequently, season length and localized geographic and temporal environmental variation are thought to play significant roles in shaping inter- and intraspecific variation in social organization of primitively eusocial insects (Richards and Packer 1996; Fucini et al. 2009; Kocher et al. 2014).

Sweat bees (Hymenoptera: Halictidae) exhibit considerable variation in social behaviour, from solitary nesting to primitive eusociality (Schwarz et al. 2007). This makes them an ideal group with which to examine the role of the environment during the early evolutionary stages of eusociality (Wcislo 1997). Eusociality in sweat bees is characterised by the presence of at least two broods: a first brood (B1) including some typically smaller female workers together with a variable proportion of males, and a second brood (B2) comprising reproductives only. It is thought that social behaviour can be expressed only where the season is sufficiently long to rear consecutive broods (Davison and Field 2016 and references therein), suggesting that sociality is temporally precluded where the season is too short (Kocher et al. 2014; but see Miyanaga et al. 1999). In at least one socially polymorphic sweat bee the expression of sociality is plastic, and the decision whether to become social may be associated with the amount of time remaining in the season after the emergence of B1 offspring (Field et al. 2010, 2012; see also Hirata and Higashi 2008). Variation in environmental conditions can strongly affect phenology, demography and colony social organisation (Packer et al. 1989; Richards and Packer 1996) by influencing the timing of nest initiation, foraging opportunities and rates of brood failure (Richards and Packer 1995; Richards 2004; Field et al. 2012; Richards et al. 2015). Furthermore, at least one socially polymorphic species is known to produce a greater proportion of B1 males in social nests situated further north (Yanega 1993). This might represent a bet-hedging strategy against the failure of B2, because mated B1 females can directly enter hibernation (Yanega 1989, 1993).

Nevertheless, it remains to be demonstrated experimentally that a shorter season length completely precludes the persistence of primitive eusociality (Kocher et al. 2014), and it is unclear to what extent apparently obligate social species are capable of exhibiting plasticity in response to novel environmental cues. For example, some unexpected behaviours are expressed only when bees are subjected to unusual conditions (e.g. Rehan et al. 2013; Quiñones and Wcislo 2015), and few studies have sought to transplant mobile taxa outside of their natural species range (Sexton et al. 2009). Previous studies of sweat bees employing field transplants have aimed to elucidate the mechanisms underpinning socially polymorphic behaviour, involving the movement of individuals between populations exhibiting alternative social phenotypes (Field et al. 2010, 2012; Davison and Field 2018; see also Cronin 2001; Baglione et al. 2002).

**Fig. 1:**
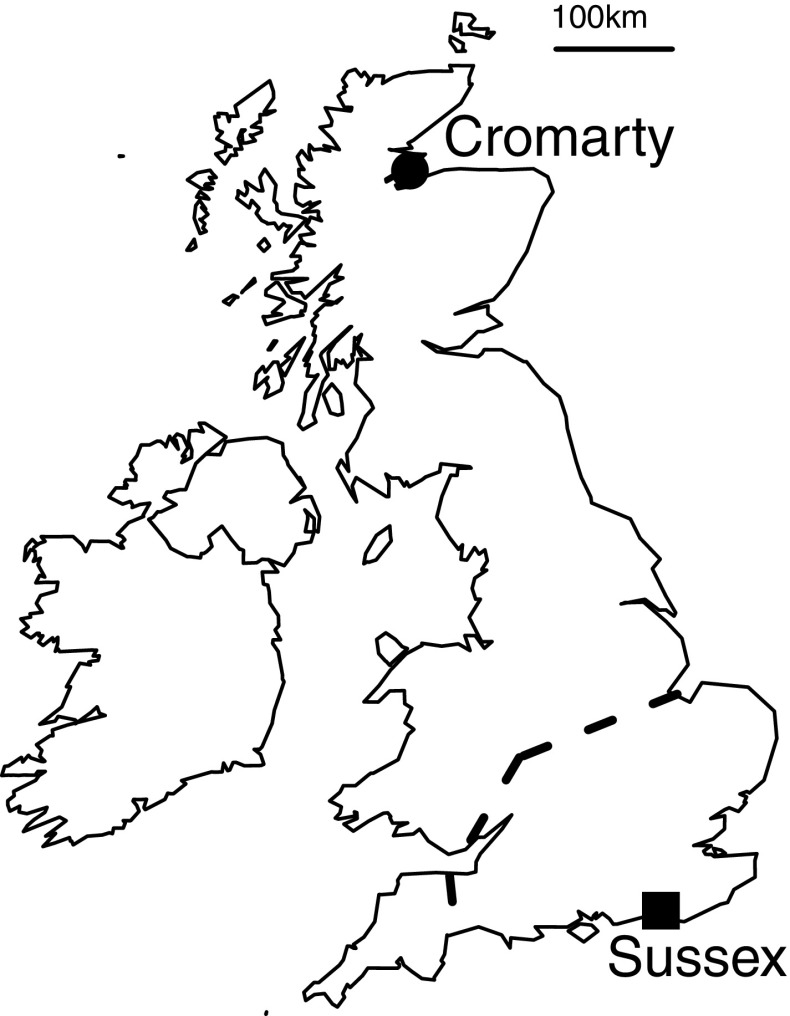
Map of the UK and Ireland showing the location of sites to which *L. malachurum* nest foundresses were transplanted. The site of the nesting aggregation from which bees were sourced was located 13 km to the east of the Sussex site, and is not shown separately in the figure. In the UK, *L. malachurum* currently persists only to the south of the dashed line

In this paper, we use field transplants to investigate the role of environmental constraints at play during the early stages in the evolution of eusociality. We transplanted the obligate primitively eusocial sweat bee *Lasioglossum malachurum* Kirby from the far south of the United Kingdom (UK) where it is known to nest socially (Packer and Knerer 1985; Davison et al. in prep.), to the far north of the UK, several 100 km further north than its recorded natural distribution (Falk 2015; Fig. 1). *Lasioglossum malachurum* is a well-studied sweat bee in which eusociality is obligate so far as is known (Wyman and Richards 2003), and with a life cycle typical of many primitively eusocial halictids. In the UK *L. malachurum* is confined to southern England (Falk 2015; Fig. 1), and the following summary of the life cycle is based on nests from this region (Packer and Knerer 1985; Davison et al. in prep.). Mated females (foundresses) emerge from hibernation and initiate subterranean nests in spring. Each female alone mass provisions a B1 of ≈ 5 sealed brood cells, each containing a single offspring and all the food required for development. B1 females emerge in summer and remain at the nest as workers, provisioning a B2 of reproductives of both sexes. Almost all B1 offspring are female (Packer and Knerer 1985 reported 2.3% B1 males) and they are on average ≈ 15% smaller than foundresses.

The fact that *L. malachurum* does not occur in the northern half of the UK suggests that transplanting it there could reveal which aspects of its biology are not adapted to cooler, northern environments, and which aspects exhibit adaptive or non-adaptive plasticity. We made three predictions for how transplantation might affect social behaviour: (1) the shorter season and cooler temperatures would delay the timing of nesting relative to control bees in the south, precluding successful B2 production; (2) due to less favourable weather conditions, foundresses would experience greater rates of both total nest failure (no B1 cells provisioned) and brood failure (death of brood after they have been provisioned); (3) transplanted foundresses might produce a more male-biased first brood. Our use of small sample sizes, and transplanting to only a single location, reduce the generality of our conclusions. Nevertheless, this study represents the first attempt to experimentally test the general prediction that primitive eusociality is temporally precluded at higher latitudes, and the first study to test the extent of behavioural plasticity in an obligate eusocial sweat bee.

## Methods

In 2013 and 2015 we transplanted *L. malachurum* foundresses from the far south to the far north of the UK (Fig. 1). This is well beyond the natural range known for obligate eusocial sweat bees in the UK and is where only solitary behaviour is expressed in socially polymorphic species (Field et al. 2012; Falk 2015; Davison and Field 2016). Nest foundresses were sourced from a substantial aggregation (≫ 1000 nests) located along a grassy footpath in the South Downs National Park, approximately 13 km to the east of the University of Sussex. Foundresses were transplanted directly from this aggregation to the University of Aberdeen’s Lighthouse Field Station at Cromarty in northern Scotland (Cromarty), with control transplants to the University of Sussex campus (Sussex) (Fig. 1).

**Fig. 2:**
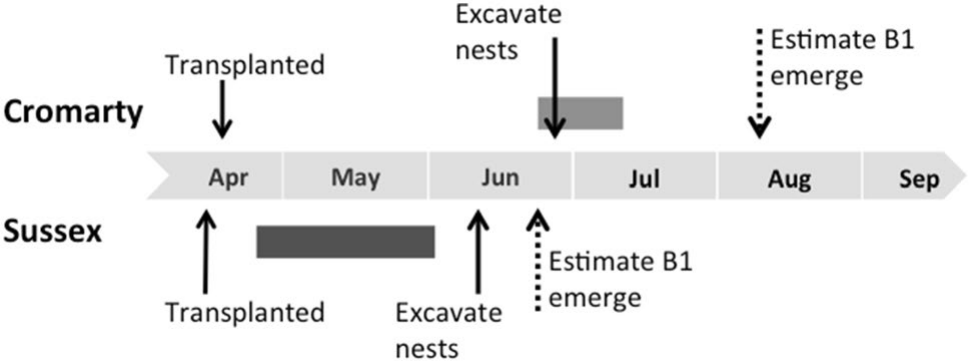
The timing of key events at Cromarty and Sussex in 2015. The dark grey bar shows the estimated period of foundress provisioning at Sussex, based on observations of *L. malachurum* nests at the source nesting aggregation. The light grey bar shows the hypothetical period of foundress provisioning at Cromarty, had nests not been excavated. See “Results” for further details of estimated foundress provisioning and B1 emergence dates

Foundresses were transplanted inside black 14L plastic buckets with drainage holes cut into the base, which were covered by fine mesh gauze to prevent bees escaping during transit. Buckets had been embedded into the ground adjacent to the nesting aggregation before nesting began in each season (*n* = 18 buckets in 2013, *n* = 78 in 2015), and were filled with compacted soil from the source nest site. These were embedded in groups of three or five at regular intervals along the entire length (ca. 80 m) of the aggregation. Buckets were kept clear from vegetation and provided bare areas of soil that foundresses naturally colonised upon emergence from hibernation during the springs of 2013 and 2015 respectively. In each year, we chose to transplant those buckets in which the most foundresses were nesting. The density of nests within a bucket does not affect the number of B1 offspring provisioned (unpublished data). During transportation, buckets were kept individually in a dark, cool environment to discourage any activity. Each was wrapped in two black plastic bin bags and placed in a sealed, padded black plastic container containing ice packs. We now describe the methods for the transplants carried out in 2013 and 2015 separately. Figure 2 details the chronology of events for the experiment conducted in 2015. Details for 2013 are not shown because both control and transplanted foundresses probably provisioned all their B1 offspring prior to transplantation, and therefore it was not possible to test our first prediction (above).

### 2013 fieldwork

#### 2013 transplant

On 31 May 2013, two buckets containing nesting foundresses and their developing B1 offspring (see below) were removed from where they had been embedded at the source nesting site, and placed in refrigerated conditions (5 °C) overnight. Over the following 2 days they were driven to Cromarty and embedded in the ground adjacent to a west-facing wall (Fig. 1). On 11 June a further two buckets were removed from the source site, refrigerated overnight, and driven around for the following 2 days as approximate controls. These were then embedded at the University of Sussex (Fig. 1).

Focal nests were not directly observed prior to transplantation in 2013. However, observations from a parallel study at the source site indicated that foundresses had ceased provisioning before buckets were removed (L. Holt pers. comm.), and no further provisioning was observed at the source nesting site after the removal of buckets used in the control transplant (JF pers. obs.). It is therefore unlikely that foundresses transplanted to Cromarty provisioned any B1 offspring after transplantation. Consequently, all B1 offspring were likely to have been provisioned at the source site. Therefore, in 2013 we focussed on only whether transplantation increased rates of brood failure, and the behaviour of B1 offspring upon emergence.

#### Nest excavations in 2013

To examine the failure rate of brood within nests, we excavated nests from one bucket each at Cromarty and Sussex prior to B1 emergence, on 04 July and 12 July respectively. In both cases foundresses were not provisioning at the time of excavation as indicated by the lack of newly provisioned brood cells. To examine whether offspring at Cromarty had successfully emerged and provisioned a second brood, the remaining bucket was excavated on 13 August, prior to the emergence of any B2 offspring. It is possible that provisioning of B2 was still ongoing at Cromarty since some nests contained living workers and small larvae. All pupae and foundresses present were recorded and stored in ethanol. We noted whether offspring were alive or dead: living larvae or pupae typically wriggle upon contact, while dead offspring often appear misshapen or squashed. In 2013 all B1 offspring were pupae, and therefore the sex of offspring was easily determined by counting the number of antennal segments (12 in females and 13 in males). After transplantation, nesting behaviour was not observed directly at either Sussex or Cromarty. The expression of eusociality at Cromarty was diagnosed by the presence of developing B2 offspring, which are most likely to have been provisioned by B1 workers. In *L. malachurum* B1 and B2 form separated cell clusters, and therefore it is easy to distinguish between B1 and B2 brood.

### 2015 fieldwork

#### 2015 transplant

Buckets were transplanted 7 weeks earlier in 2015 than in 2013. Observations made at the source site prior to transplantation confirmed that foundresses did not begin provisioning before either control or treatment buckets were removed. Therefore, in 2015 all foundresses provisioned their B1 offspring at the site to which they were transplanted, so that we obtained data from all stages of the nesting cycle. Four buckets were removed from the source nesting site on 10 April and transplanted to Sussex as controls for transplantation itself. A further four buckets were removed on 16 April and embedded in the ground at Cromarty on 18 April, in exactly the same location as buckets transplanted in 2013. During transit, buckets were treated the same as in 2013. As in 2013 nests were not directly observed at either Sussex or Cromarty after transplantation, and the expression of eusociality was determined by the presence of B2 brood (see above).

#### Nest excavations in 2015

Due to transplantation occurring much earlier in 2015, we additionally examined total nest failure, productivity and phenology at Sussex and Cromarty. Two buckets were excavated prior to B1 emergence on 10–12-June and 24–25-June at Sussex and Cromarty respectively. We excavated nests at Cromarty later because we expected nesting to have been delayed relative to Sussex. At time of excavation, it was not clear that foundresses at Cromarty had finished provisioning in two nests that contained living foundresses, since all nests contained very young larvae. During excavations of B1 offspring at Cromarty, five nests were excavated but one of these collapsed before the age of offspring could be scored. However, the number of provisioned cells could still be counted because it was possible to detect the completed provision masses. Nests were excavated for B2 offspring on 06 August and 08 September at Sussex and Cromarty respectively. As in 2013, all larvae, pupae and adults were recorded and stored in ethanol. As a measure of foundress provisioning effort, we noted the number of cells that had been provisioned (i.e. contained pollen) regardless of whether they contained developing offspring.

#### Brood genotyping in 2015

All B1 offspring excavated at Cromarty were larvae, which cannot be sexed visually, and we determined their sex using microsatellite genotyping (see Parsons et al. 2017 for methodology and further details). Larvae were genotyped at eight loci and were designated as haploid males when only a single allele was detected at each locus amplified (Table S1). One locus failed to amplify across all individuals, and so data from seven loci only are presented in Tables S1, S2. Because we were interested in only the sex of brood, we scored individual larvae on gels as either homozygous (one band) or heterozygous (two bands) for each locus. We detected two B1 males, one each in two nests at Cromarty (see “Results”), neither of which amplified across all eight loci (see Table S2). The probability of scoring a homozygous female by chance for these two individuals was 0.001 and 0.012 respectively (Table S2).

### Climate and weather data

**Fig. 3:**
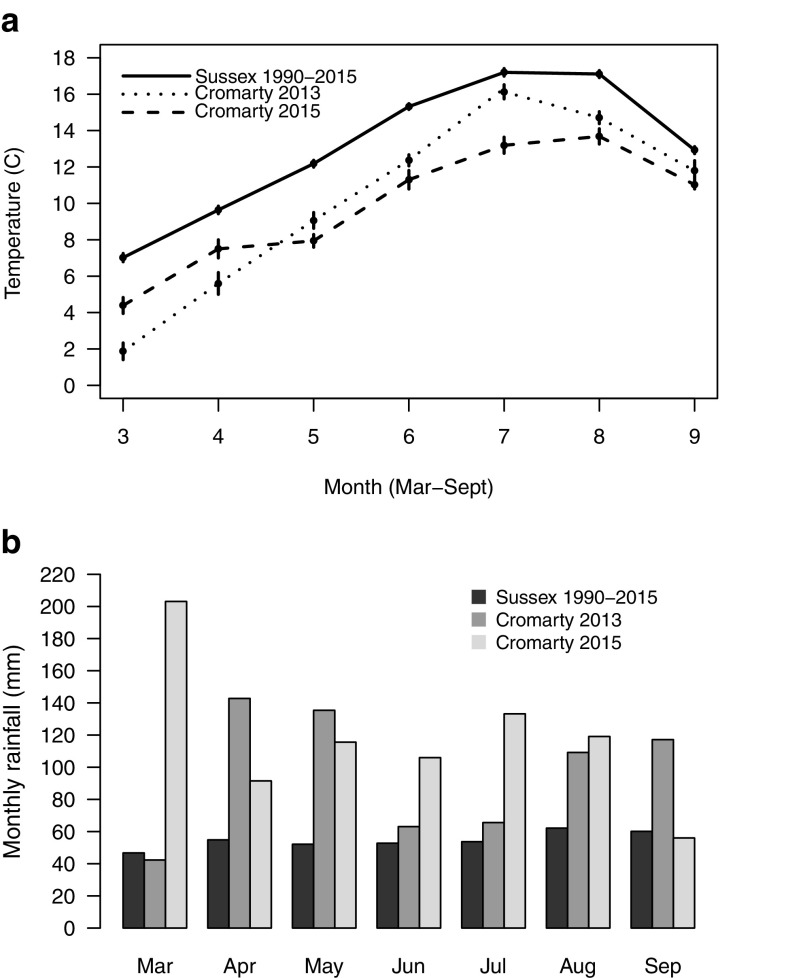
**a** Mean monthly temperatures for southeast England, representing “Sussex” (solid line, long-term average 1990–2015), and Cromarty in 2013 (short dashes) and 2015 (long dashes). **b** Monthly rainfall for southeast England, representing “Sussex” (black bars, long-term average 1990–2015), and Cromarty in 2013 (grey bars) and 2015 (light grey bars). Temperatures are presented ± SE

To provide a baseline for conditions typically experienced by *L. malachurum* at the source nesting site, we constructed a 25 years time series of mean monthly temperature and rainfall for the southeast of England (Fig. 3), where *L. malachurum* is most prevalent in the UK. Data covering 1990–2015 were downloaded from the UK Meteorological Office website (http://www.metoffice.gov.uk/climate/uk/summaries/2015/October/regional-values). To examine how conditions experienced by bees transplanted to Cromarty in both 2013 and 2015 deviated from those typically experienced by *L. malachurum*, temperature data were downloaded from a nearby web-based weather station located at Inverness Airport (http://www.wunderground.com). Localised rainfall data were not available; therefore, we used regional monthly rainfall values for northern Scotland from 2013 to 2015 respectively as indicative of conditions at Cromarty in both years, downloaded from the UK Meteorological Office website.

### Data analysis

We hypothesised that the phenology of bees transplanted to Cromarty in 2015 would be delayed relative to bees at Sussex. To examine this, we scored the age of living B1 offspring excavated from nests as follows: pollen ball = 0, very small larva = 1, small larva = 2, medium larva = 3, large larva = 4, white pupa (wp) = 5, wp brown eyes = 6, wp black eyes = 7, pigmented pupa = 8. One of the nine nests excavated at Sussex in 2015 contained only a single dead B1 offspring, which could not be aged and therefore we analyse data from only eight nests. The bimodal structure of the ‘brood age’ data in 2015 meant we could not analyse it using a generalised linear mixed model (GLMM), and as such we were unable to account for ‘nest ID’ or ‘bucket ID’. Therefore, we averaged the age scores within nests and used the Mann–Whitney–Wilcoxon test to examine differences in age between brood excavated at Cromarty and Sussex. Brood age data came from just a single bucket at Cromarty because the other bucket contained the nest that collapsed upon excavation, and we did not detect a significant difference in age between offspring from the two buckets excavated at Sussex (Mann–Whitney–Wilcoxon test: *p* = 0.14, *n* = 4 nests in each bucket). Although brood did not spend their whole development at Cromarty in 2013, we also scored the age of B1 offspring to test whether being transplanted to Cromarty significantly slowed their development relative to control bees at Sussex. We analysed this data in the same way as for 2015.

To compare Cromarty and Sussex in 2015 in terms of the rate of nest failure, and in terms of the number of cells provisioned during the B1 stage (including or excluding completely failed nests), we used GLMMs with binomial and negative binomial errors respectively. Because two buckets were excavated at each site, we included ‘bucket ID’ as a random factor. Nests were considered to have failed only if they contained no provisioned B1 cells. B1 offspring excavated at Cromarty in 2015 were significantly younger than those at Sussex (see “Results”). To avoid artificially inflating the failure rate at Sussex (brood will have had more time during which to fail) we used the number of cells that had been provisioned (i.e. inclusive of those that had failed) to compare the number of potential B1 offspring provisioned by foundresses between sites. Analyses of the number of B1 offspring and nest failure rates from 2015 thus focus on foundress provisioning opportunities. We additionally used a generalised linear model (GLM) with binomial errors to test whether transplantation to Cromarty in 2013 resulted in a greater proportion of brood failing than at Sussex. Due to the small expected sample sizes, we used Fisher’s exact test to examine whether foundresses were more likely to be excavated alive alongside B1 offspring at Cromarty or Sussex in both 2013 and 2015.

All analyses were conducted in the *R* environment (R Development Core Team 2013), and the MASS package (Venables and Ripley 2002) was used for performing the GLM with negative binomial errors. We used the lme4 package (Bates et al. 2015) to perform GLMMs. Results are presented ± 1 standard error.

All data generated or analysed during this study are included in this published article and its supplementary materials.

## Results

### Prediction 1

Phenology will be delayed at Cromarty relative to control transplants at Sussex, and preclude the successful rearing of B2 offspring.

In 2013, B1 offspring were excavated from nine nests at Cromarty and 20 nests at Sussex. There was no difference in the age of offspring (all pupae) excavated from Cromarty and Sussex (Mann–Whitney–Wilcoxon test *W* = 91.5, *p* = 0.233; *x̅* B1 age at Sussex = 6.14 ± 0.09, *x̅* B1 age at Cromarty = 5.86 ± 0.14). B2 offspring were excavated from 11 nests at Cromarty in 2013 (*x̅* = 3.82 ± 0.84 B2 offspring per nest), demonstrating that in these nests B1 females emerged and behaved as workers. Excavations were not conducted for B2 at Sussex in 2013. However, observations conducted over several years show that B1 females always behave as workers at the source nesting site and at Sussex.

**Fig. 4:**
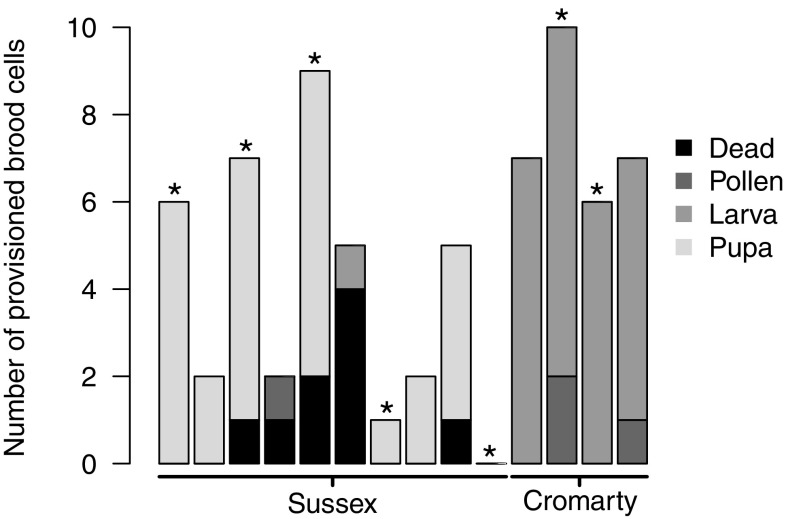
The number of B1 brood cells provisioned by foundresses transplanted to Sussex and Cromarty in 2015, and the age of brood within each nest (see legend). Each column represents a different nest. Note than an additional nest at Cromarty containing four provisioned brood cells is not shown because the age of brood could not be determined (see “Methods”)

In 2015, B1 offspring excavated prior to their maturation at Cromarty were significantly younger (all larvae) than B1 offspring at Sussex (all pupae), despite being excavated nearly 2 weeks later (Fig. 4; Mann–Whitney–Wilcoxon test *W* = 32, *p* = 0.006, $\bar {x}$B1 age at Cromarty = 1.59 ± 0.36, *n* = 4 nests; Sussex = 5.9 ± 0.42, *n* = 8). To estimate the difference in phenology between the two sites, we conservatively assumed that control foundresses began provisioning 2 weeks after being transplanted to Sussex. This is justified because by this time (23 April) *L. malachurum* foundresses had been observed provisioning at the source site (C. Couchoux pers. comm.). The pupal stage typically comprises about one-third of total development time in sweat bees (see table 14-2 in Yanega 1997), and we estimated that when they were excavated, B1 offspring at Sussex had approximately 1 week of development remaining. Thus, we took 17 June as their predicted date of eclosion, giving an estimated development time of 50 days, or about 7 weeks. This is in line with development times previously reported for *L. malachurum* (Weissel et al. 2006). Most offspring excavated at Cromarty were only very small larvae, not more than a week old. We therefore conservatively assume that most offspring at Cromarty were provisioned 1 week prior to excavation (18 June), about the same time as B1 emergence at Sussex. Thus, we estimated that the life cycle at Cromarty was approximately 7 weeks delayed relative to Sussex. Note that our estimates of development time are conservative, because Weissel et al.’s (2006) study was from a warm region in central Europe.

When estimating the likely emergence date of B1 offspring at Cromarty, we note that mean temperatures during June, July and August at Cromarty in 2015 never greatly exceeded typical May temperatures at Sussex (Fig. 3a). Because higher temperatures later in the season would not therefore have accelerated development, we conservatively assume that Cromarty B1 offspring would have had a similar development time (53 days) to B1 offspring at Sussex. Therefore, if B1 offspring at Cromarty had survived to eclosion, we estimate they would not have emerged until around 9 August, again approximately 7 weeks later than B1 offspring at Sussex.

There was no evidence of eusociality at Cromarty in 2015. Of 14 nests initiated in buckets that were transplanted to Cromarty and excavated in September, none contained any B2, and therefore failure to produce B2 offspring at Cromarty was 100%. Nest excavations aimed at locating B2 offspring at Cromarty revealed the presence of B1 cells and some pollen in the soil, but no attempt to construct or provision B2 cells. This suggests that foundresses had provisioned B1 offspring, as found in the two buckets excavated prior to B1 maturation, but that these offspring failed to eclose. Moreover, the only evidence of adult bees at Cromarty was a single foundress-sized head buried in the soil. In contrast, at Sussex 12 nests were excavated before B2 emergce, and six (50%) contained provisioned B2 offspring ($\bar {x}$ = 8.33 ± 2.30, range 3–19). The similarity of this failure rate to the rate of nests failing to produce any B1 offspring at Sussex (see below), suggests that few additional nests failed completely once workers had emerged.

### Prediction 2

Foundresses transplanted to Cromarty will suffer increased rates of failure to provision any B1 cells, successful foundresses will provision fewer B1 offspring, and a greater proportion of their brood will fail.

In 2015, 19 nests were initiated each in the buckets transplanted to Cromarty and Sussex respectively, which were excavated before the emergence of B1 offspring. Although bees at Cromarty did suffer a higher failure rate overall, there was no significant difference between Cromarty and Sussex in the proportion of foundresses that failed to provision any B1 offspring [GLMM: *X*^2^_1_ = 1.091, *p* = 0.296, Cromarty = 14/19 (74%) failed, Sussex = 10/19 (53%) failed]. Because of this high rate of failure, some of our sample sizes are small. Nevertheless, considering nests in which B1 brood cells were provisioned, foundresses transplanted to Cromarty provisioned slightly more brood cells than foundresses transplanted to Sussex, although this difference was not significant (Fig. 4; GLMM: *X*^2^_1_ = 2.867, *p* = 0.091, Cromarty = 6.80 ± 0.97, *n* = 5 nests; Sussex = 4.33 ± 0.91, *n* = 9). Moreover, when nests containing no provisioned brood cells (i.e. completely failed) were included, this trend disappeared and there was no difference in the number of brood cells provisioned (GLMM: *X*^2^_1_ = 0.036, *p* = 0.850, Cromarty = 1.79 ± 0.68, Sussex = 2.05 ± 0.65). In 2013, brood at Sussex and Cromarty were the same age because they had been laid before transplanting, so that it was possible to test whether transplantation to Cromarty directly affected rates of brood failure. There was no difference in the proportion of dead brood in nests excavated at Cromarty or Sussex (GLM: *X*^2^_13_ = 0.70, *p* = 0.403). Foundresses were also equally likely to be found alive in their nests during excavations for B1 offspring in 2013 (Fisher’s exact test: *p* = 0.205; foundresses detected at Cromarty = 4/9, Sussex = 15/20) and in 2015 (Fisher’s exact test: *p* = 1; foundresses detected at Cromarty = 2/5, Sussex = 5/9).

### Prediction 3

Foundresses transplanted to Cromarty will lay a greater proportion of B1 male eggs.

Sex ratios were considered from 2015 only because foundresses provisioned offspring prior to transplantation in 2013. At Sussex, no males were detected in the nine nests that were excavated and contained B1 offspring, and therefore each foundress produced a 100% female first brood. The sex of offspring could be determined at only four of the five nests excavated at Cromarty: two produced 100% females and two produced a single male each (80 and 89% female-biased in each case).

## Discussion

Harsh or unpredictable environmental conditions are thought to play a key role in promoting social behaviour across a range of taxa (Jetz and Rubenstein 2011; Faulkes and Bennett 2013; Kocher et al. 2014). However, some of the benefits of sociality may not apply to primitively eusocial insects in harsh environments because their annual life cycle could be temporally precluded or prove too risky (Kocher et al. 2014; Fu et al. 2015). We investigated environmental barriers to the evolution of eusociality by transplanting the primitively eusocial sweat bee *L. malachurum* far to the north of its natural range boundary (Fig. 1). We showed that when B1 females produced by Cromarty-transplanted foundresses reached adulthood in 2013, they still behaved as workers and provisioned B2 offspring. However, in 2015 when foundresses were transplanted at the start of the season and despite provisioning as many B1 offspring as in south-east England, provisioning was significantly delayed such that it would not be possible to successfully rear B2 offspring (Figs. 2, 4). There was also some limited evidence that transplanted foundresses may have responded by producing a few B1 male offspring. We now discuss our results in relation to our three main predictions. In doing so, we note that without transplanting more bees, and without transplanting to other northern or high-altitude locations, we cannot rule out the possibility that small sample sizes, or environmental factors peculiar to Cromarty, have had an influence.

### Nesting phenology

Foundresses transplanted to Cromarty in 2015 began nesting approximately 7 weeks later in the season than control-transplanted bees, and it is highly unlikely that B1 females would have emerged early enough to successfully rear a B2 (Figs. 2, 4). Although our transplant was limited to a single location, our results provide the first experimental support for the idea that primitive eusociality is absent from high altitude or latitude communities because the season is too short to rear consecutive broods (Soucy and Danforth 2002; Fucini et al. 2009; Kocher et al. 2014; Davison and Field 2016). Activity levels in small bees such as *L. malachurum* are positively correlated with ambient temperature (Bishop and Armbruster 1999; Schürch et al. 2016), which at Cromarty lagged far behind the long-term average for the southeast UK. For example, temperatures at Cromarty as late as June and July did not exceed those recorded much earlier, in May, at Sussex (Fig. 3a). Foundresses provisioning B1 was delayed concomitant with the lag in temperature (Figs. 2, 3a), suggesting that environmental factors at Cromarty placed significant constraints on the timing of provisioning (Potts 1995). Field at al. (2012) recorded shifts in spring phenology and social phenotype after reciprocally transplanting the socially plastic sweat bee *Halictus rubicundus* Christ. Since *L. malachurum* is an obligate eusocial species, however, our data demonstrate that the timing of provisioning changed in response to abiotic factors, rather than as part of a strategic shift to non-sociality.

Primitively eusocial sweat bees tend to have shorter egg to adult development times than non-social bees (Kocher et al. 2014). Theoretically, faster development or the rearing of smaller offspring in response to cooler conditions could compress the life cycle and enable eusociality in sweat bees to persist further north (Nylin and Gotthard 1998; Gotthard et al. 2000; Inagawa et al. 2001). However, although foundresses from more northerly populations of sweat bees tend to be smaller, there is no evidence for increased growth rates during immature development (Field et al. 2012; Davison and Field 2017, 2018). Most intraspecific variation in development time is apparently driven by temperature, such that development is prolonged at lower temperatures (Weissel et al. 2006; Field et al. 2012); moreover, sweat bees can exert only limited control over temperatures experienced by developing brood, such as by locating nests in exposed, south-facing ground (Potts and Wilmer 1997; Hirata and Higashi 2008). Thus, future climate warming is predicted to increase the northerly range of obligate eusocial sweat bees (Schürch et al. 2016): indeed, within the last 25 years *L. malachurum* has rapidly expanded its range northwards within the UK and become much commoner (Falk 1991, 2015).

Unlike most other bees, sweat bees must reach adulthood, mate, feed and enter hibernation in the year that they are born (Michener 2007). Although this may sometimes provide a head start in spring (Matthews 1991), it places more severe constraints on the timing of laying B2 eggs. Despite exhibiting annual life cycles and mating before hibernation, bumblebees are able to extend the active season because they can regulate their body temperatures independently of the environment (Heinrich 1979). Consequently, bumblebees can persist even into the Arctic Circle (Martinet et al. 2015); far exceeding the northern range limits of eusocial sweat bees. Some solitary sweat bees persisting in harsh environments reproduce in multiple years (e.g. Field 1996), and some extreme high latitude solitary insects circumvent the short growing season by completing development over multiple years (Varpe 2017). The only known perennial eusocial sweat bee, *L. marginatum*, produces a single brood of workers each year for up to 5 years before rearing a final brood of reproductives (Plateaux-Quénu 1962). Such a strategy might seem well suited to enduring short seasons yet *L. marginatum* is restricted to warm climates (Pesenko et al. 2000), perhaps in part because the extremely delayed production of reproductives is too risky in unpredictable environments.

### Foundress provisioning and brood failure

Our prediction that Cromarty-transplanted foundresses would provision fewer B1 offspring and suffer higher rates of brood failure were only partially supported. In 2015, foundresses transplanted to Cromarty provisioned the same number of B1 cells as control foundresses at Sussex and experienced the same rate of failure to provision at all, although our sample sizes were small. Moreover, in 2013, there was no difference between Cromarty and Sussex in the rate of B1 brood failure. However, in 2015 it is likely that no B1 offspring at Cromarty survived to emergence, and therefore that nest failure was 100%. In comparison, B1 offspring successfully provisioned a B2 in 50% of control nests at Sussex. It is perhaps surprising, given the considerable delay in phenology and thus presumably prolonged period of inactivity before foraging, that foundresses transplanted to Cromarty in 2015 did not experience greater rates of nest failure or provision fewer B1 offspring. Because foundresses lose around 90% of their pre-hibernation fat reserves by the end of the season (Weissel et al. 2012), however, it may be that any early differential costs incurred by foundresses at Cromarty would have become apparent only later during a B2 phase.

*Lasioglossum malachurum* foundresses appear to provision similar numbers of B1 offspring across European populations (Packer and Knerer 1985; Paxton et al. 2002; Strohm and Bordon-Hauser 2003; Davison et al. in prep; this study). This number was not perturbed by transplantation (Fig. 4), suggesting that provisioning opportunities were not more limited at Cromarty than Sussex. As mass provisioners, sweat bees probably need only a single day to provision each offspring (Richards 2004), and thus can capitalise on available days of suitable weather (e.g. Field 1996). In contrast, Inagawa et al. (2001) showed that in a progressively provisioning paper wasp, more northerly foundresses produced fewer workers and hence fewer reproductives. *Lasioglossum malachurum* is also polylectic (Westrich 1989; Polidori et al. 2010), which may have enabled Cromarty-transplanted foundresses to more readily utilise available resources. However, we were unable to compare the quantity of pollen provided to B1 offspring at Cromarty and Sussex. If Cromarty-transplanted foundresses experienced fewer or shorter suitable foraging windows, or fewer resources overall, they may have provisioned an equal number of smaller offspring (Richards and Packer 1996; Richards 2004, but see Richards et al. 2015). Indeed, adult sweat bees do tend to be smaller in more northerly environments (Field et al. 2012; Davison and Field 2017).

Cromarty experienced exceptional levels of rainfall in 2015 (Fig. 3b; Thompson pers. comm.), coinciding with the total failure of Cromarty-transplanted foundresses to successfully rear any B1 offspring to adulthood. Extreme inter-year variability in weather conditions is common in northern environments (e.g. Packer et al. 1989), and ground-nesting Hymenoptera can be particularly susceptible to heavy and persistent rainfall even after provisioning has occurred (Packer 1992; Richards and Packer 1995; Davison and Field 2016). First brood cells of *L. malachurum* are arranged in a cluster surrounded by a partial cavity, which presumably functions to aid drainage (Sakagami and Michener 1962; Packer and Knerer 1986; Packer 1991). However, it may be that *L. malachurum* remains poorly adapted to such high levels of rainfall because it typically inhabits dry regions (Pesenko et al. 2000), and is notably absent from the wetter southwest of the UK (Falk 2015; Fig. 1).

### Sociality and behavioural plasticity

B1 offspring appeared to exhibit little plasticity in response to transplantation. In 2013, B1 females reached adulthood at Cromarty and provisioned a B2 as normal, also demonstrating that conditions at Cromarty do not always preclude worker activity. In Central Europe, *L. malachurum* does exhibit plasticity in the number of worker broods produced, although the precise mechanism remains elusive (Weissel et al. 2006). Our results, however, suggest that *L. malachurum* cannot strategically omit the worker brood altogether. There was limited evidence that Cromarty-transplanted foundresses increased the proportion of B1 males. No males were detected in any nests in which B1 offspring were provisioned either at the source nesting site (all nests in 2013) or at Sussex (controls in 2015), and B1 males have been recorded only exceptionally rarely in our on-going studies of *L. malachurum* in the southern UK (Davison et al. in prep; see also Packer and Knerer 1985). Nevertheless, single males were detected in two out of the four nests excavated at Cromarty in 2015. It is possible that these males were produced in response to cues associated with increased day length at the time of oviposition (e.g. Yanega 1993, 1997), and had B1 offspring emerged, could have enabled females to enter hibernation. However, although mated workers are detected in *L. malachurum* nests with varying frequency, they do not routinely enter hibernation (Wyman and Richards 2003) so that potential fitness gains through increased male production are uncertain.

## Conclusion

Our results provide experimental evidence that season length, together with poor weather conditions at more northerly latitudes, place a proximate constraint on the evolution and geographic distribution of eusociality in sweat bees. *L. malachurum* may have exhibited limited plasticity in response to transplantation by producing a small number of B1 males, but the season at Cromarty is likely to normally be too short and conditions too variable between years to allow the persistence of eusociality in the absence of derived adaptations such as pereniality or nest thermoregulation (Packer et al. 1989; Richards and Packer 1996; Hoiss et al. 2012; Kocher et al. 2014; Fig. 3). Two important limitations of our study were firstly that Cromarty could have been a poor site for *L. malachurum* for unknown reasons independent of season length; and secondly that our sample sizes were small. To extend our work, it would therefore be interesting to transplant more bees to multiple northern or high-altitude sites, thus subjecting bees to a range of microclimates and levels of resource availability.

## Electronic supplementary material

Below is the link to the electronic supplementary material.

Supplementary material 1 (DOCX 13 KB)

Supplementary material 2 (XLSX 12 KB)

Supplementary material 3 (DOCX 20 KB)
