# Supplementary material for: Environmental barriers to sociality in an obligate eusocial sweat bee
Source: Insectes Soc. 2018 Jul 4;65(4):549–59. doi: 10.1007/s00040-018-0642-7 (PMC6208632; doi:10.1007/s00040-018-0642-7)
Supplement: Supplementary file 1 — Supplementary material 1 (DOCX 13 KB) [file 40_2018_642_MOESM1_ESM.docx]

Supplementary data explanatory notes

The supplementary data file contains the data that we collected and analysed during the study and is presented in the paper. For each column, rows represent individual nests. Columns A-O show data gathered and analysed in 2015 from Cromarty and Sussex, and columns Q-W data collected at Cromarty and Sussex in 2013.

There is a discrepancy in the number of nests for which data on the age, number and sex ratio of B1 offspring at Cromarty in 2015 are reported. This is because during nest excavations, one of the nests collapsed before the age of brood could be determined. The brood (likely to have been at an early stage and thus very small) were lost. However, the provision masses were still clearly visible in the soil, and so it was possible to count the number of offspring provisioned (number of provision masses) but not determine their age or sex. Therefore, B1 age (column A) and sex ratio (columns K-O) are reported for only four nests, whereas >0 B1 offspring are reported from for five nests at Cromarty (column F). Age is also reported from only eight nests at Sussex in 2015(column F) whereas brood number is reported from nine (column C). This is because one nest contained a single provisioned B1 offspring, which was dead and could not be aged.

Note that for the number of B2 offspring produced at Cromarty in 2013 (column S), we did not know the number of nests originally initiated in the bucket. Therefore, we do not know the number of nests that failed to produce B2 offspring.
