# Supplementary material for: Environmental barriers to sociality in an obligate eusocial sweat bee
Source: Insectes Soc. 2018 Jul 4;65(4):549–59. doi: 10.1007/s00040-018-0642-7 (PMC6208632; doi:10.1007/s00040-018-0642-7)
Supplement: Supplementary file 3 — Supplementary material 3 (DOCX 20 KB) [file 40_2018_642_MOESM3_ESM.docx]

| Individual | LMA14 | LMA51 | LMA36 | LMA52 | LMA02 | LMA53 | LMA03 | Sex |
| --- | --- | --- | --- | --- | --- | --- | --- | --- |
| 291 | 2 | 1 | 1 | 2 | 1 | 2 |  |  |
| 292 | 2 | 1 | 1 | 1 | 2 | 2 |  |  |
| 293 | 2 | 1 | 1 | 2 | 2 | 2 |  |  |
| 294 | 2 | 1 | 1 | 1 | 2 | 2 |  |  |
| 295 |  | 2 |  | 2 | 2 | 2 |  |  |
| 297 |  |  | 2 | 2 | 1 |  |  |  |
| 298 | 2 |  | 1 | 2 | 2 | 2 |  |  |
| 299 | 2 | 2 | 2 | 2 | 1 | 1 |  |  |
| 300 | 2 | 1 | 1 | 2 | 2 | 2 |  |  |
| 301 | 2 |  | 1 | 2 | 2 | 2 |  |  |
| 302 |  | 1 | 1 | 2 |  | 1 |  |  |
| 303 |  |  | 2 | 2 | 1 |  |  |  |
| 304 | 2 | 2 | 1 | 2 | 2 | 1 |  |  |
| 305 |  | 1 |  | 1 | 1 | 1 | 1 | MALE |
| 307 | 1 | 1 | 1 | 2 | 2 | 2 |  |  |
| 308 |  |  |  |  |  |  |  |  |
| 309 | 1 |  | 1 | 2 | 2 | 2 |  |  |
| 310 | 1 |  | 2 | 2 | 2 | 2 |  |  |
| 311 | 1 | 1 | 2 | 2 |  | 2 |  |  |
| 312 |  |  | 2 | 2 |  |  |  |  |
| 313 |  |  |  |  |  |  |  |  |
| 314 | 1 |  | 1 | 1 |  |  |  | MALE |
| 315 |  |  | 2 | 2 |  |  |  |  |
| 316 |  |  | 1 | 2 |  |  |  |  |
| 317 |  |  | 1 | 2 |  |  |  |  |
| 318 |  | 2 | 2 | 2 |  | 1 |  |  |
| 319 |  |  | 2 | 2 |  | 2 |  |  |
| 320 |  |  | 1 | 2 |  | 2 |  |  |

Table S1 Details of the genotype scores for brood from Cromarty in 2015. ‘1’ signifies homozygote, ‘2’ signifies heterozygote. Blank cells are missing data. Since male sweat bees are diploid, they will appear as homozygote across all amplified loci. See Table S2 for calculations showing the chances of a female scoring homozygous at all loci by chance in the two males identified below

Table S2 Heterozygosity and numbers of alleles detected for the microsatellite markers used in the present study ^a^, and the probability of a diploid female being homozygous by chance for the two individuals (bottom two rows, shown in bold) detected as B1 males at Cromarty in 2015. Missing allele scores are designated by ‘NA’. Note that the two males were from separate nests.

|  | LMA14 | LMA51 | LMA36 | LMA52 | LMA02 | LMA53 | LMA03 | Probability of female Ho^b^ by chance^c^ |
| --- | --- | --- | --- | --- | --- | --- | --- | --- |
| No. alleles | 14 | 4 | 8 | 5 | 10 | 9 | 7 | – |
| Hobs^d^ | 0.825 | 0.475 | 0.737 | 0.732 | 0.87 | 0.675 | 0.816 | – |
| **1-HObs** | **0.175** | **NA** | **0.263** | **0.268** | **NA** | **NA** | **NA** | **0.0123347** |
| **1-HObs** | **NA** | **0.525** | **NA** | **0.268** | **0.130** | **0.325** | **0.184** | **0.001093802** |

^a^ The *L. malachurum* females used to develop these microsatellite loci originated from the same nesting aggregation as all bees in the present study, and the loci amplified in the present study show no evidence of linkage disequilibrium (Parsons et al. 2017)

^b^ Homozygote

^c^ Calculated by multiplying the 1-HObs value for each scored locus together

^d^ Observed heterozygosity
